# Eliminate now: seven critical actions required to accelerate elimination of *Plasmodium falciparum* malaria in the Greater Mekong Subregion

**DOI:** 10.1186/s12936-016-1564-3

**Published:** 2016-10-21

**Authors:** Andrew A. Lover, Roly Gosling, Richard Feachem, Jim Tulloch

**Affiliations:** 1 Malaria Elimination Initiative, Global Health Group, University of California, San Francisco, USA; 2 Independent consultant, Cali, Colombia

## Abstract

The emergence in 2009 of *Plasmodium falciparum* parasites resistant to the primary therapies currently in use (artemisinin-based combination therapy, ACT) in Southeast Asia threatens to set back decades of global progress in malaria control and elimination. Progress to date through multiple sets of initiatives and partners to contain or eliminate these parasites has been hampered due to a wide range of organizational, financial, and health systems-level challenges. In this commentary, a set of seven specific and concrete actions are proposed to directly address these issues and to accelerate *P. falciparum* elimination within the Greater Mekong Subregion to avert a wider public health crisis. These actions are specifically needed to elevate the situation and response mechanisms to those of a true emergency; to address systems-level challenges with personnel limitations and stock-outs of key commodities; and to restructure the response mechanisms to be well-aligned with the required outcomes. Consideration of these issues is especially pressing with planning meetings for renewal of the Regional Artemisinin-resistance Initiative (RAI) framework slated for late 2016 and into 2017, but these suggestions are also relevant for malaria programmes globally.

## The problem

The emergence in 2009 of *Plasmodium falciparum* parasites resistant to the primary therapies currently in use (artemisinin-based combination therapy, ACT) in Southeast Asia threatens to set back decades of global progress in malaria control and elimination. With the passing of the 3-year anniversary of the unveiling of the WHO emergency response to artemisinin resistance (ERAR) within the Greater Mekong region (GMS) [1], the time is ideal to reflect upon the challenges ahead, and to propose a set of critical actions for rapid progress (Table 1).

**Table 1:** Critical actions for rapid elimination of *Plasmodium falciparum* in the Greater Mekong Subregion

Declare a Public Health Emergency of International Concern (PHEIC)
Establish a command-and-control structure
Eliminate stock-outs of ACT medicines and RDTs at peripheral sites
Consider realistic time-limited incentives for elimination fieldwork
Provide dedicated leadership support to national malaria programmes
Massively strengthen face-to-face support to peripheral levels
Track *operational* progress via indicators that capture essential field activities

A comprehensive review of artemisinin-resistance containment efforts in 2012 stated that the initial response to AR was ‘good, but delayed’, and concluded: ‘*For effective regional action convening of meetings and passing of resolutions and declarations is not enough. Responsible entities need the mandate and resources to follow*-*up to ensure that agreements are implemented*.’ [2].

**Fig. 1:**
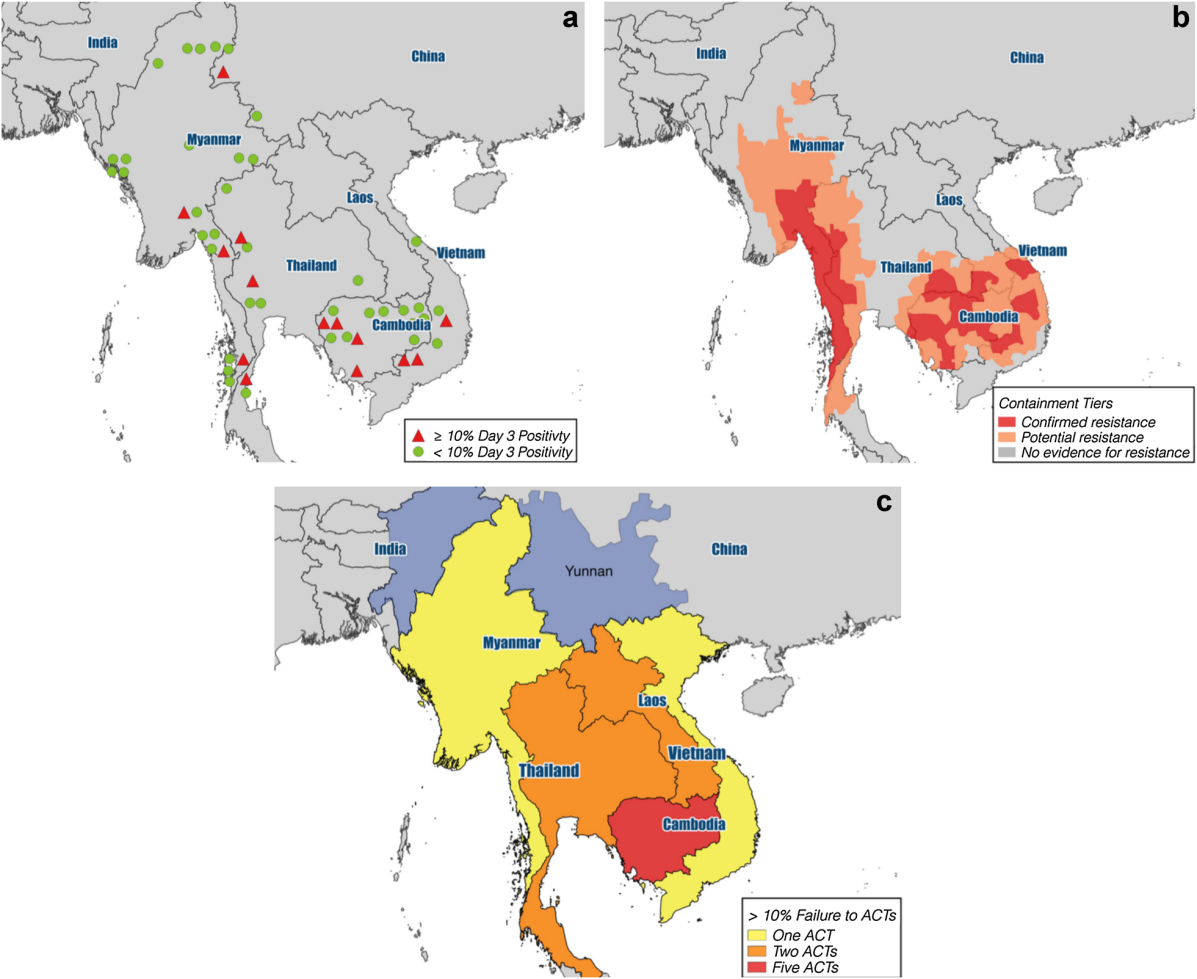
Geographic spread of reported artemisinin resistance in the Greater Mekong Subregion (GMS); reporting dates 2011 (**a**); 2014 (**b**); and 2015 (**c**). (Adapted from WHO updates [35])

The initial control efforts within the Thai-Cambodia border region around the town of Pailin (the epicentre of resistance) were not successful, and containment of these parasites to border regions of Myanmar, Vietnam, Thailand, Lao PDR, Cambodia and China (Yunnan province) is unfeasible due to multiple populations of resistant parasites. Evidence suggests the parasite mutates under intense drug pressure, and this has occurred at multiple sites independently within the GMS [3]; the genetic markers of artemisinin resistance (specific Kelch-13 mutations) have now been identified throughout Myanmar [4] and now encroach upon the Indian subcontinent (Fig. 1). Finally there is increasing resistance to multiple partner drugs, including dihydroartemisinin-piperaquine [5, 6].

Consequent to these new challenges, the campaign has expanded to one of *elimination* of all *P. falciparum* parasites within the region [7]. For *P. falciparum* elimination to occur, a credible full scale ‘war’ on the parasite has to be undertaken [8]. However, for this campaign to be successful, multiple unaddressed challenges need to be comprehensively considered, and measures to address them rapidly applied to strategic planning.

In any planning to avert a large-scale public health crisis, the need for rapid implementation must be balanced against the demand for sustainable health systems strengthening. This tension is unavoidable in the current emergency response; system-building (inherently a gradual and long-term process) must remain a focus, but a secondary one in the planning for elimination of *P. falciparum* in the region.

## Seven critical actions

This commentary does not attempt to design a comprehensive framework for malaria elimination; WHO and its partners provide that guidance. Instead, it highlights seven actions that are critical to accelerating malaria elimination efforts. Although it is motivated by the urgent need to more effectively address multi-drug resistant (MDR) *P. falciparum* in the GMS, it is hoped that it will provoke discussion not only of these issues for the region, but also of the requirements for malaria elimination globally.

## Declare a Public Health Emergency of International Concern (PHEIC)

The PHEIC mechanism as developed and implemented by the WHO allows the organization to formally declare that a situation is “*an extraordinary public health event which is determined to constitute a public health risk to other countries through the international spread of disease; and to potentially require a coordinated international response*.” [9] Zika virus disease was declared a PHEIC in February 2016 on the basis of limited epidemiological data [10]. It is somewhat difficult to reconcile this action with the current situation in the GMS; MDR-*P. falciparum* certainly poses an equally grave international threat. There is also somewhat of a disconnect between the use of the word ‘Emergency’ in the title of the ERAR programme, and the outcomes from previous WHO-mediated debates on the potential for declaring AR a public health emergency, where it was determined that the situation did not rise to the level of a PHEIC.

While it must be acknowledged that the actions necessary to address MDR-*P. falciparum* are inherently different in nature from prior PHEICs (e.g. responses to Ebola virus disease), the potential impacts are equally dire. While current evidence suggests resistance parasite strains are not evolutionarily competitive in sub-Saharan Africa [11], it would be prudent to assume that this will not remain the case. If or when these parasites spread to sub-Saharan Africa, the world can expect major impacts. Evolution of resistance to chloroquine is estimated to have caused two- to six-fold increases in malaria mortality and morbidity throughout sub-Saharan Africa [12], and modelling suggests the potential for ACT failures to cause 10 to 78 million additional malaria cases over a 4-year period [13].

There should be no doubt that the response to MDR-*P. falciparum* needs to be funded, approached, and managed as an emergency. If the current stringent criteria necessary to declare a PHEIC are not met by the MDR-*P. falciparum* threat, we suggest an alternative categorization should be created to elevate such ‘slow-motion’ emergencies to the proper level of global attention: perhaps a ‘Category II PHEIC’? The elevation of MDR-*P. falciparum* to a PHEIC, or creation of an additional PHEIC classification to cover situations such as AR, will require member states to actively work with WHO in lobbying for these changes.

## Establish a command-and-control structure

The current response to MDR-*P. falciparum* has been hampered by fragmentation and the bureaucratic nature of all coordination and oversight. Responsibility is spread between multiple WHO offices (Geneva-based Headquarters; regional offices for South-East Asia and Western Pacific Regions; and the ERAR Hub in Cambodia), each country’s NMCP, plus funding and actions involving multiple partners, especially under the Global Fund supported Regional artemisinin-resistance initiative (RAI). Additionally, the multi-stakeholder Regional Steering Committee of the $100 million USD RAI has no mandate or authority to direct or coordinate actions across all dimensions of response to MDR-*P. falciparum* in the GMS. This situation is in stark contrast to the end stages of smallpox eradication [14], and more recent efforts to eliminate polio or tackle SARS [15]. In all of these programmes, there has been a single unified structure under WHO coordination, with clear lines of responsibility and accountability.

To achieve the levels of coordinated action required to confront MDR-*P. falciparum*, there should be a single regional coordinator, elected and empowered by a multi-stakeholder governing body that includes representation of the GMS NMCP directors, with a broad managerial and financial mandate. Responsible and *accountable* focal points must then be identified at country and provincial levels. The overall role and mandate of this structure should be clearly defined, and fully supported by existing regional structures, including WHO and the Asia–Pacific Leaders Malaria Alliance (APLMA) and the Asia–Pacific malaria elimination network (APMEN), but there must be a single locus of coordination for a well-organized, flexible and comprehensive response. The necessity for such an approach in eliminating (then “eradicating”) malaria in the GMS was specifically highlighted as early as 1959:

“*Men, money and materials must be welded into* ***an organization*** *that has the resources to achieve its objects and is flexible enough to meet the unforeseen difficulties that must be expected in any biological struggle*” [16] (emphasis added).

Creating a new and parallel body would only complicate the regional landscape further, and an organization built around existing technical and managerial capacity in the region, significantly strengthened by secondments from appropriate institutions, that would supersede the current mechanisms is envisioned. This would require all partners to defer authority to the designated regional coordinator, which will require a combination of diplomacy and selflessness. However, the potential impacts of MDR-*P. falciparum* are such that the governance and structure of the response demand creativity and true collaboration unimpeded by “turf battles.”

Finally, the critical impact of a charismatic and driven leader to advocate, ‘push’ and motivate (as DA Henderson was in the smallpox eradication campaign) was highlighted decades ago: “*Apart from having technical knowledge,* [the eradication programme director] *should be a malariologist of great experience and administrative ability, and a man* [sic] *of character and a leader*” [17]. The response to MDR-*P. falciparum* will require a torchbearer to advocate at the highest levels; and the role of diplomat and mediator should also not be discounted, especially in light of the highly complex regional politics.

## Eliminate stockouts of RDTs and ACT medicines

Despite improvements in commodity supply with the ERAR, the response to MDR-*P. falciparum* has been hampered by stock-outs of critical point-of-care diagnostics and drugs [18]. RDTs and ACT medicines must be continuously stocked at all district and village-level facilities in malarious areas, even during the rainy season with all the logistical challenges that this entails. This deceptively simple activity is the bedrock of effective case management, and the impact of motivated and incentivized village-level malaria workers has been clearly demonstrated in multiple countries in the region [19, 20]; however, these highly effective workers can only have impact when adequate supplies are available [21]. Most national malaria control programmes (NMCPs) in the region have limitations in stock-level tracking and management, and so organizations with specialists in supply chain management should be supported to form a consortium of partners with key expertise and incentivized to directly assist with logistics and commodity distribution wherever needed, towards achieving “zero stock-outs.”

## Consider realistic time-limited incentives for elimination field work

Malaria elimination requires that health workers (both public sector and non-government) are willing and empowered to go beyond their routine activities. This requires efforts to go the extra distance to find and investigate cases, apply additional rigour in interventions, and to report with complete accuracy. They need to *want* to eliminate malaria; achieving this is in part a question of incentives, both monetary and otherwise. The economic realities of staff on the ground need to be realistically considered, especially in light of the modest salaries for many government health staff, some of whom are obliged to have other sources of income to provide a reasonable standard of living.

The considerable funding flowing into the region for malaria (projected to be ~$150 million USD in 2016) has potentially distorted the incentive system in a manner that is unsustainable in the long run, and there are clear reasons why donors are unwilling to subsidize government sector salaries. However this should be balanced by evidence that incentives, whether salary top-ups or simply covering out-of-pocket expenses for fieldwork, have produced results [22, 23]; and withdrawing them at a time when malaria elimination requires a surge of activity will almost certainly not galvanize action.

Addressing this dilemma may require innovative solutions, potentially including subsidizing the formation of truly integrated vector-borne disease programmes that nevertheless maintain malaria-specific capabilities; or creation of scholarship programmes for more junior staff working on malaria elimination. Direct payment of bonuses to teams of workers for producing well-defined and measurable results should also not be ruled out; and payment could even take the form of contributions to staff pension schemes. Implementation of these incentive programmes will require close collaborations between Ministries of Health, Finance, and Planning in each of the GMS countries, which should be started as rapidly as possible.

Elimination programmes have been specifically highlighted as an important opportunity to stimulate and energize health sector staff towards overall health systems strengthening [24, 25], and this should be fully harnessed. Secondly, local solutions need to be urgently developed that incentivize NMCPs at all levels to make the extra efforts required knowing this will likely transition their jobs, and even their programmes, out of existence [26]. Acknowledgement and transparent planning around this transition is critical to support staff morale, and rapid progress.

## Provide dedicated support to NMCP leaders in countries

There is pressing need for direct leadership support within the NMPCs. The limited number of experienced malariologists in NMCP offices face a difficult dilemma: they must remain fully engaged in high-level national and regional dialogues, but also be available for the leadership and management of practical day-to-day malaria activities. It is often that latter that suffers, hampering regional progress. One potential avenue to address this issue is externally funded support for programme and technical management. This could, for example, consist of two full-time support positions within each GMS country- one dedicated to policy and technical issues, the other for finances and on-the-ground project management, but needs would vary in each programme. The sole responsibility of these positions would be to support the national and provincial level NMCP management on *practical* aspects of malaria elimination- the size and scope of necessary response to MDR-*P. falciparum* is such that NMCPs leaders need far greater support.

## Massively strengthen face-to-face support to peripheral levels

The variability in motivation and engagement of health facility and village-level malaria staff has been highlighted as a major concern [2, 27]. While tangible incentives are important in this regard, so too is knowing that higher levels in the organizational structure are engaged in, and fully support, field workers’ efforts. This requires creation of a well-oiled ‘machine’ to organize, supply, support and supervise operations of these staff, and this is as equally important for the critical village level workers (whether paid or otherwise) as it is for other health staff.

Deployment of technical elimination teams who spend their time *in the field* at district and provincial sites would address this gap; indeed, this structure was mandated in the global malaria eradication campaign (GMEP) in India [28]. Malaria will not be eliminated from offices in capital cities, or without the direct involvement and support of in-country staff. Currently, NMCPs do not have ready access to adequate and flexible funding to support regular fieldwork, and too many administrative responsibilities to get out of their offices-supportive supervision cannot be a special activity to be carried out only infrequently.

It is proposed that these field support staff would be full-time positions supporting sub-national managers, conducting problem-solving with fixed field staff, monitoring programme implementation, and providing direct and constant feedback to the NMCP. Support of this nature was specifically highlighted as a contributing factor to smallpox eradication:

“*The quality and nature of supervision were of vital importance. The best results were obtained where WHO, national, and state or provincial supervisory staff travelled frequently into the field to review activities and to work with field staff in resolving problems*” [29].

The smallpox eradication programme used a combination of international staff and national staff redeployed from their usual duties for several months to fill these supervision roles under WHO coordination; this model could be followed for malaria elimination. Partner organizations already working on malaria elimination could be asked to provide field staff to help manage programme logistics and supportive supervisions to ‘free-up’ NMCP staff to focus on targeted elimination activities. Additionally, a cadre of highly motivated and energetic field staff could be recruited through donor-country volunteer programmes, international and national NGOs, and the private sector to work alongside redeployed government staff for field-based support.

A second facet of this issue is a need for detailed consideration of the true scale of the necessary response; two illustrative comparisons suggest that a considerable scale-up of human resources for malaria elimination is needed. In the 1930’s the Rockefeller Foundation-led elimination of *Anopheles gambiae* (*arabiensis*) from Brazil required 4000 field staff working full-time over 22 months to eliminate the vector from around 45,000 km^2^ [30]; and during the GMEP, India had programme units covering about 1 million population, *each* of which had four trucks, two jeeps, and two pickups, and 250 total staff [31]. While population-level indoor residual spraying of all households is a different type of programme from current elimination activities, these numbers highlight the sheer magnitude of effective programmes, and if activities like weekly fever surveys, or mass drug administration are needed, then comparable levels of staffing and transport must be considered.

## Track *operational* progress via indicators that capture essential field activities

The major metrics for regional malaria elimination focus on indicators like morbidity, mortality, and bed-net coverage (e.g., Appendix 3 in [1]). However, additional metrics need to be aligned with Leonard Bruce-Chwatt’s view of malariology as a field of ‘blood, mud and sweat’ [32]. The critical and largely ignored gap is one of tracking the activities of muddy boots on the ground, and in end-of-the road clinics, villages and informal settlements. Malaria elimination requires intensive effort at the farthest periphery of the health sector, and well beyond. Comprehensive malariometric indicators and measurement of interventional coverage is certainly the ideal, but may not be realistic at the furthest periphery in often difficult conditions [33]. These “bottom-line” measures will not be achieved without good management of actions extending to the most remote cases. Knowing if and how these actions are taking place is possible and moreover, critical to success, as specifically emphasized in the 1940’s during Brazil’s Yellow Fever programme:

“*Even after it was apparent that* A. aegypti *could be eradicated from any given area with the technique described, final results were often disappointingly slow and it was found that success in species elimination was to be achieved only on the basis of careful organization and meticulous supervision”* [34].

## Conclusions

This commentary makes the case that malaria elimination, and more specifically the elimination of MDR-*P. falciparum* in the GMS, will not be achieved without investments that go beyond those needed to maintain routine NMCP activities. All successful disease elimination programmes to date have involved very significant support to organize, support and oversee effective field operations. There is every reason to assume malaria elimination will be no different.

With respect to MDR-*P. falciparum* in the GMS time is short, and rapid consideration of major policy changes is critical. It is imperative that the next wave of investment is applied with utmost attention to effectiveness and efficiency; this implies strategic changes and applying lessons from successful historical programmes. The anticipated funding by the Global Fund of the all-important RAI being discussed now and into early 2017, as well as potential new investments from other donors, provides a critical opportunity to honestly assess what will be required to accelerate elimination of multi-drug resistant *P. falciparum* and to invest resources accordingly. Failure to do so will put in question not only the outcome of that effort, but also the seriousness of the global health community’s commitment to malaria elimination.
